# Tunable Magnetic Heating in La_0.51_Sr_0.49_MnO_3_ and La_0.51_Dy_0.045_Sr_0.445_MnO_3_ Nanoparticles: Frequency- and Amplitude-Dependent Behavior

**DOI:** 10.3390/nano15090642

**Published:** 2025-04-23

**Authors:** Mourad Smari, Monica Viorica Moisiuc, Mohammad Y. Al-Haik, Iordana Astefanoaei, Alexandru Stancu, Fedor Shelkovyi, Radel Gimaev, Julia Piashova, Vladimir Zverev, Yousef Haik

**Affiliations:** 1 Center for Advanced Materials Research, Research Institute of Sciences and Engineering, University of Sharjah, Sharjah P.O. Box 27272, United Arab Emirates; 2 Faculty of Physics, Alexandru Ioan Cuza University of Iasi, 700506 Iasi, Romania; monica_moisiuc@yahoo.com (M.V.M.);; 3 Department of Sustainable and Renewable Energy Engineering, University of Sharjah, Sharjah P.O. Box 27272, United Arab Emirates; 4 Faculty of Physics, M.V. Lomonosov Moscow State University, Leninskie Gori, 119991 Moscow, Russia; 5 Faculty of Mechanical Engineering, University of Ljubljana, Aškerčeva 6, 1000 Ljubljana, Slovenia; 6 Department of Mechanical and Nuclear Engineering, College of Engineering, University of Sharjah, Sharjah P.O. Box 27272, United Arab Emirates; 7 Department of Mechanical Engineering, The University of Jordan, Amman 11942, Jordan; 8 BIMAI-Lab, Biomedically Informed Artificial Intelligence Laboratory, University of Sharjah, Sharjah P.O. Box 27272, United Arab Emirates

**Keywords:** magnetic hyperthermia, perovskite manganite nanoparticles, specific absorption rate (SAR), ferrofluid viscosity, nanoparticle heating efficiency

## Abstract

The use of perovskite manganite nanoparticles in magnetic hyperthermia has attracted significant attention due to their tunable magnetic properties and high specific absorption rate (SAR). In this work, we present a combined experimental and theoretical investigation of the frequency- and amplitude-dependent magnetic heating behavior of La_0.51_Sr_0.49_MnO_3_ (LSMO) and Dy-doped La_0.51_Dy_0.045_Sr_0.445_MnO_3_ (DLSMO) nanoparticles. The nanoparticles were synthesized via the sol–gel method and characterized by XRD and SEM, while SAR values were experimentally evaluated under varying magnetic field strengths (60–120 Oe) and frequencies (150–300 kHz). In parallel, theoretical modeling based on Néel and Brownian relaxation mechanisms was employed to predict SAR behavior as a function of particle size, magnetic anisotropy, and fluid viscosity. The results reveal that Dy doping enhances magnetic anisotropy, which modifies the relaxation dynamics and leads to a reduction in SAR. The model identifies the optimal nanoparticle size (~18–20 nm) and ferrofluid viscosity to maximize heating efficiency. This combined approach provides a comprehensive framework for designing and optimizing perovskite-based nanoparticles for magnetic hyperthermia applications.

## 1. Introduction

Traditional methods of treating cancerous tumors include chemotherapy and radiation therapy, but scientists and medical professionals are focused on finding alternative methods that are less harmful to the patient health. Magnetic hyperthermia is less invasive than surgical intervention and does not cause nausea and fatigue, unlike chemotherapy. The main advantage is the possibility of localized cancer therapy, especially for deeply located, difficult-to-reach tumors. Heating of the tumor also causes a significant increase in the temperature of adjacent healthy tissues. Possible side effects resulting from hyperthermia treatment may include overheating, blood clot formation, burns, and cardiovascular disorders. Magnetic hyperthermia relies on biocompatible magnetic nanoparticles that generate heat through magnetic losses upon exposure to an alternating electromagnetic field, leading to controlled thermal ablation of tumor cells. The therapeutic temperature window for effective hyperthermia ranges from 38 °C to 46 °C, where mild heating enhances immune responses, while temperatures exceeding 43 °C trigger irreversible cellular damage [1,2,3,4]. Simultaneous increases in the frequency and amplitude of the alternating electromagnetic field lead to an undesirable increase in eddy currents and their side effects on healthy tissues. In contrast, their impact on the circulatory system (blood rheology) and other body systems is poorly understood. To avoid their adverse effects, the Brezovich criterion was proposed: the product of the amplitude of the magnetic field strength and the frequency should not exceed ~10^9^ A/(m.s). Thus, the safe confirmed level of the alternating magnetic field in medicine lies in the range of 7.5–15 kA/m (90 ÷ 180 Oe) with a frequency of 50–100 kHz and is determined by the Brezovich criterion [5]. The heating efficiency of magnetic nanoparticles is primarily governed by their size, magnetic anisotropy, and Curie temperature (*T_C_*) [6,7]. Bulk ferromagnetic particles exhibit low specific absorption rate (*SAR*) values due to their large coercive fields and significant eddy current losses, limiting their applicability in biomedical settings [8,9]. In contrast, superparamagnetic nanoparticles efficiently convert electromagnetic energy into heat under moderate field strengths, offering tunable *SAR* values while mitigating aggregation-related complications [10,11]. The transition from ferromagnetic to superparamagnetic behavior, dictated by the critical size threshold, is crucial in ensuring colloidal stability and biocompatibility. Magnetic susceptibility determines the heating capability of single-domain particles. As particle size increases, hysteresis losses become more significant, and the extent of heating is governed by the hysteresis loop area, which depends on saturation magnetization and coercive force [12,13]. For even larger particles, viscous friction effects become noticeable due to their movement in the medium. The heat dissipation of a nanoparticle is directly linked to its ability to absorb energy from the magnetic field, which is characterized by the specific absorption rate (*SAR*) [14,15]:

(1) $$SAR=C\frac{dT}{dt}\frac{M}{m}$$

where *C* is the heat capacity of the magnetic fluid, ΔT is the temperature increase over the heating interval (t), and M/m is the ratio of the mass of water to the mass of nanoparticles.

The primary parameter limiting the heating of multidomain ferromagnetic nanoparticles is the *T_C_*. If the *T_C_* falls within the optimal hyperthermia range, the material remains a strong candidate for further investigation. However, nanoparticles transition into a superparamagnetic state at a certain particle size, where the blocking temperature (*T_B_*) becomes the dominant factor. Typically, lower than the Curie temperature, *T_B_* governs the maximum achievable heating, as observed in La_0.75_Sr_0.25_MnO_3_ nanoparticles with an average diameter of 20.9 nm [16]. A hysteresis loop in these nanoparticles suggests heat losses due to remanent magnetization, even though, theoretically, superparamagnetic particles should not exhibit hysteresis. This phenomenon arises from low remanent magnetization and the coexistence of larger particles [17]. The blocking temperature for this composition is 335 K. In contrast, the Curie temperature is 367 K. Notably, increasing the amplitude of the external magnetic field beyond a threshold does not further enhance the maximum heating temperature. The cessation of heating near the blocking temperature can be explained by the relationship between field amplitude and the heating rate (dT/dt), which initially increases but does not significantly alter the final temperature (ΔT). The time-dependent heating profile is expressed as follows [18]:

(2) $$T(t)=T_{0}+\Delta T\cdot \left(1-\exp(-t/\tau_{R}\right))$$

where T_0_ is the initial temperature and τ_R_ is the relaxation constant, dependent on the heat capacity, particle surface properties, and heat transfer coefficient between the particles and the surrounding medium. This model effectively describes heating and cooling behavior under low magnetic fields (<110 Oe); however, as *T_B_* is approached, deviations from exponential relaxation occur [19,20]. The *SAR* is typically quadratically dependent on the applied field amplitude for superparamagnetic particles, where relaxation-based heating dominates over hysteresis losses. Experimental approximations of the *SAR* (*H*) using a power function yielded an exponent of *a* = 2.4, indicating that the heating mechanism is not solely relaxation-based but includes hysteresis contributions. Further studies on La_0.75_Sr_0.25_MnO_3_ have shown that heating efficiency increases with particle size, peaking at 49 nm under a magnetic field of 88 mT and a frequency of 108 kHz [21].

Perovskite manganite, particularly La_1−x_Sr_x_MnO_3_ (LSMO) compositions, has garnered interest due to its intrinsic magnetic properties, including tunable *T_C_*, high saturation magnetization, and excellent chemical stability. By adjusting the Sr and La ratios, the magnetic response and heating efficiency can be tailored to optimize hyperthermic performance while preventing excessive heating that may compromise surrounding healthy tissues [22,23,24]. Doping strategies such as Dy incorporation offer additional control over the magnetic phase transition and enhance the field-driven thermal response [25,26,27,28]. This study investigates the frequency- and amplitude-dependent heating behavior of La_0.51_Sr_0.49_MnO_3_ and La_0.51_Dy_0.045_Sr_0.445_MnO_3_ nanoparticles. We systematically evaluate the *SAR* and thermal response under varying field conditions and elucidate the interplay between particle size, magnetic anisotropy, and dynamic field parameters. These insights provide a pathway for optimizing perovskite-based nanoparticles for precision hyperthermia applications.

## 2. Experimental Procedure and Characterization Techniques

The sol–gel method was used to synthesize nanometric manganite powders with compositions of Dy0.00 and Dy0.045. High-purity, analytical-grade reagents were used as precursors. Lanthanum nitrate (La(NO_3_)_3_·6H_2_O), strontium nitrate (Sr(NO_3_)_2_), and manganese nitrate (Mn(NO_3_)_3_·4H_2_O) were sourced from Sigma-Aldrich (St. Louis, MO, USA), while dysprosium oxide (Dy_2_O_3_) was utilized as the Dy precursor. The synthesis began with precise weighing of the precursors, followed by dissolution in 200 mL of distilled water under continuous stirring at 75 °C for 1 h under ambient conditions. To facilitate the dissolution of Dy_2_O_3_, a few drops of nitric acid were introduced. A molar ratio of 1:1 between the metal ions and citric acid was maintained, ensuring effective chelation. Ethylene glycol was subsequently added, achieving a final metal ion: citric acid: ethylene glycol ratio of 1:1:2. The mixture was homogenized for 3 h before being gradually heated to 150 °C, allowing evaporation to progress until gel formation. The resulting gel was then dried at 300 °C overnight. The final annealing process was carried out in three steps: 600 °C for 12 h, 800 °C for 12 h, and 1000 °C for 12 h in an air atmosphere. Stepwise annealing temperatures of 600 °C, 800 °C, and 1000 °C were chosen to sequentially remove organic precursors, initiate crystallization, and optimize grain growth and magnetic properties. Previous studies have shown that such controlled annealing significantly influences grain size, crystallinity, and magnetic behavior, including MS and TC, by improving the structural coherence and reducing defects at grain boundaries [29,30].

## 3. Characterization Techniques

The crystalline structure of the synthesized samples was analyzed using X-ray Powder Diffraction (XRD) with an Empyrean PANalytical diffractometer (PANalytical, Almelo, Overijssel, The Netherlands) equipped with a Cu Kα1 radiation source (λ = 1.54056 Å) in Bragg–Brentano θ-2θ geometry. Phase identification was performed using the PDF-01-085-6112 reference database. The microstructural features were investigated via Scanning Electron Microscopy (SEM) using a Jeol JSM-7100F field emission gun (FEG) microscope (FE-SEM, JSM-7100F; JEOL Ltd., Tokyo, Japan). operated at 15 kV in secondary electron (SE) mode to capture high-resolution images of the sample morphology.

## 4. Structural and Morphological Analysis

**Figure 1:**
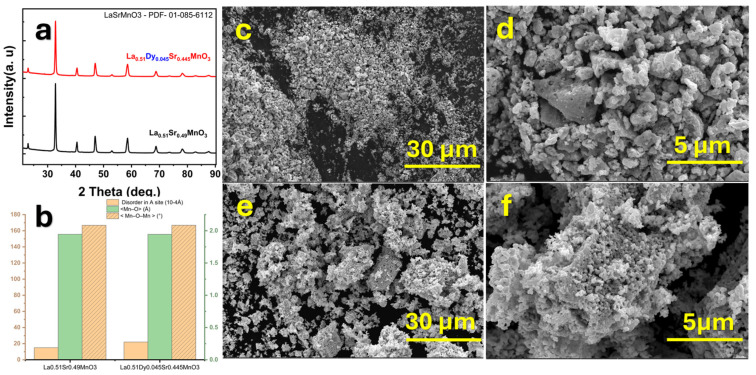
(**a**) X-ray diffraction (XRD) patterns of La_0.51_Sr_0.49_MnO_3_ and Dy-doped La_0.51_Dy_0.045_Sr_0.445_MnO_3_, showing phase identification using Cu Kα radiation (λ = 1.5406 Å). (**b**) Schematic representation illustrating changes in lattice disorder and Mn–O–Mn bond angles. (**c**–**f**) SEM images of the nanoparticle morphology taken at 15 kV, showing porosity and grain-size evolution with Dy doping.

The structural properties of La_0.51_Sr_0.49_MnO_3_ (Dy0.00) and La_0.51_Dy_0.045_Sr_0.445_MnO_3_ (Dy0.045) were investigated using X-ray diffraction (XRD), as shown in Figure 1a. The diffractograms confirm the formation of the perovskite phase, indexed to the orthorhombic structure with the Pnma (62) space group, consistent with the standard PDF-01-085-6112 pattern. The substitution of Dy results in slight peak shifts, indicating modifications in lattice parameters due to variations in ionic radii. The disorder at the A site and the Mn–O–Mn bond angles are influenced by Dy incorporation, as depicted in Figure 1b. With Dy doping, a reduction in the lattice parameters (b and c), along with a decrease in cell volume, is observed [31]. This confirms that Dy^3+^ substitution at the La^3+^ site leads to structural distortions that alter the Mn–O bond lengths and Mn–O–Mn angles, affecting the electronic and magnetic interactions in the material. Figure 1c–f present SEM images of the samples at different magnifications. The micrographs reveal a porous and agglomerated morphology, which is characteristic of sol–gel-derived perovskite oxides. The Dy0.00 sample (Figure 1c,d) shows larger, loosely packed grains, while Dy0.045 (Figure 1e,f) exhibits a denser and more interconnected microstructure, suggesting enhanced grain growth and improved crystallinity. The higher magnification images (Figure 1d,f) further highlight the effect of Dy doping, where smaller and more uniform grains are evident in Dy0.045. These morphological differences may impact the material’s electronic and catalytic properties by altering the surface area and intergranular connectivity. The SEM images indicate that Dy-doped LSMO nanoparticles exhibit a finer and more uniform distribution than the undoped LSMO. This microstructural refinement can be attributed to the Dy^3+^ ions, which introduce local strain fields that hinder excessive grain growth during annealing. The reduction in grain size is consistent with the SAR trends, where Dy-doped samples show a shift in optimal heating efficiency toward slightly smaller particle sizes (~18 nm vs. ~20 nm). This suggests that the improved size uniformity enhances magnetic relaxation dynamics, supporting more effective energy dissipation under alternating magnetic fields.

## 5. Theoretical Estimates of the SAR

In this model, the values of the SAR were computed considering only Néel and Brownian relaxations; these types of nanoparticles are in the superparamagnetic regime. Néel relaxation depends on the magnitude of the magnetic anisotropy barrier energy that must be present for the magnetic moment of a particle to reverse its direction. The value of this energy depends on the anisotropy of the particle (material, shape) and the volume of the nanoparticle. The Brownian relaxation mechanism depends on the ability of a particle to reverse the direction of magnetization only due to the physical rotation of the particle in a physiological solution, which depends on two parameters: the viscosity of the liquid and the hydrodynamic diameter of the particle [15,32].

The volumetric heating rate generated by a single nanoparticle (*P*, W/m^3^) and the imaginary component of the magnetic susceptibility (*χ*″) can be expressed based on Néel and Brownian relaxation mechanisms as follows [33,34]:

(3) $$P=\mu_{0}f\pi \chi^{\prime \prime }H_{0}^{2}\mathrm{and}\;\chi^{\prime \prime }=\frac{\mu_{0}M_{s}^{2}V}{{3k}_{B}T}\frac{2\pi f\tau }{1+{\left(2\pi f\tau \right)}^{2}}$$

where *H*_0_ (kA/m) is the intensity of the magnetic field, *f* (kHz) is the frequency of the magnetic field, $\mu_{0}=4\pi \cdot 10^{-7}$ H/m is the permeability, $M_{s}$ (kA/m) is the saturation magnetization, and $k_{B}=1.38\times 10^{-23}J/K$ is the Boltzmann constant. These relations depend strongly on the particle size; the field parameters (frequency (*f*) and field amplitude (*H*_0_, kA/m)); and effective relaxation time τ, which contains the Brownian relaxation time ($\tau_{B}$) and Néel relaxation time $\tau_{N}$:

(4) $$\tau =\frac{\tau_{N}\tau_{B}}{\tau_{N}+\tau_{B}},\tau_{B}=\frac{{3\mu V}_{H}}{k_{B}T};\;\tau_{N}=\tau_{0}\frac{\sqrt{\pi }}{2}\frac{exp\left[\frac{KV}{k_{B}T}\right]}{\sqrt{\frac{KV}{k_{B}T}}}$$

where $\tau_{0}$ is the average relaxation time, μ is the viscosity of the carrier liquid, $V_{H}=V{\left(1+\frac{\delta }{R}\right)}^{3}$ is the hydrodynamic volume of the particles, δ is the thickness of the surfactant layer, *K* is the anisotropy constant, $V=\frac{4\pi R^{3}}{3}$ is the volume, and R is the radius of the nanoparticle. The SAR of magnetic nanoparticles is governed by multiple factors, including the following:

1. The frequency and amplitude of the applied magnetic field;
2. The nanoparticle size;
3. The viscosity of the ferrofluid.

All material parameters, including particle size (*d*), anisotropy constant (*K*), saturation magnetization (*Ms*), and ferrofluid viscosity (μ), have a strong influence on Néel and Brownian relaxation mechanisms and, implicitly, on *SAR* values. Brownian relaxation time ($\tau_{B}$) is strongly size-dependent. Néel relaxation time ($\tau_{N}$) is also indirectly influenced by this parameter (*d*). A larger volume of the nanoparticle leads to a longer Néel relaxation time and potentially less effective heating at high frequencies. The anisotropy constant (*K*) influences the energy barrier for Néel relaxation. A higher value of *K* leads to slower Néel relaxation and shifts the optimal *SAR* frequency.

The saturation magnetization (*Ms*) influences *SAR* directly. Higher values of Ms increase the SAR. Brownian relaxation is strongly ferrofluid viscosity-dependent. Higher values of viscosity lead to slower particle rotation and longer relaxation times ($\tau_{B}$), corresponding to slower Brownian relaxation. This mechanism becomes faster for lower values of ferrofluid viscosity. Better efficiency of magnetic relaxation results in more energy dissipated as heat per unit time. Consequently, *SAR* values increase. When $\tau_{B}$ increases due to higher values of viscosity, the Brownian relaxation mechanism dominates, the effective relaxation time increases, and the *SAR* decreases accordingly with relation (4).

**Figure 2:**
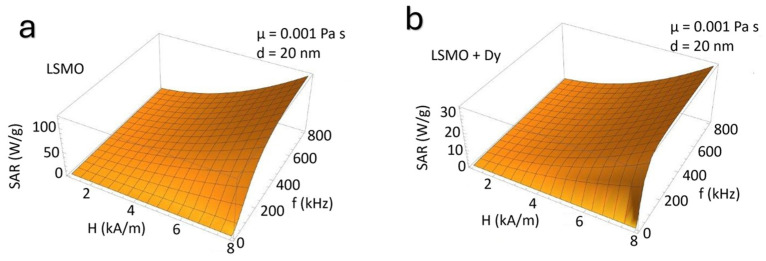
(**a**) SAR (W/g) of LSMO nanoparticles as a function of magnetic field amplitude (H, KA/m) and frequency (f, kHz). (**b**) SAR variation under identical conditions for Dy-doped LSMO nanoparticles. Measurements were conducted in aqueous dispersion at room temperature.

Figure 2 presents the variation of the *SAR* as a function of the applied magnetic field amplitude (*H*) and frequency (*f*) for both undoped and Dy-doped La_0.51_Sr_0.49_MnO_3_ (LSMO). In Figure 2a, the *SAR* of LSMO exhibits a pronounced increase with rising field strength and frequency, demonstrating an expected enhancement due to the interplay between Néel and Brownian relaxation mechanisms. In contrast, Figure 2b shows that Dy doping significantly reduces the *SAR* values under comparable conditions. This reduction suggests that Dy incorporation modifies the intrinsic magnetic properties, altering the anisotropy energy barrier and relaxation dynamics. The observed trends underscore the crucial role of doping in tailoring the hyperthermia performance of manganite-based nanoparticles [35,36].

**Figure 3:**
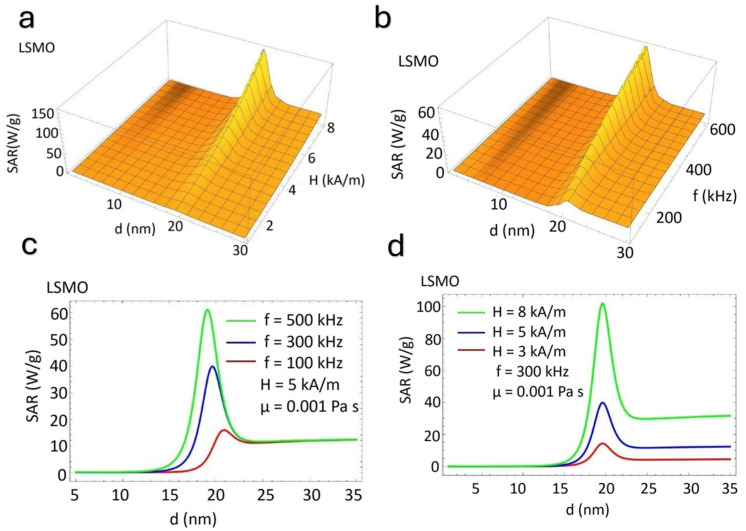
(**a**,**b**) Three-dimensional surface plots and (**c**,**d**) two-dimensional contour plots showing the SAR (W/g) of LSMO nanoparticles versus particle size (d, nm), magnetic field amplitude (H, KA/m), and frequency (f, kHz). Experiments were conducted with 10–30 nm particle sizes under fields of 60–120 Oe.

Figure 3 illustrates the dependence of the SAR on the nanoparticle size (*d*) for LSMO under varying magnetic field amplitudes and frequencies. In Figure 3a,b, three-dimensional surface plots highlight a pronounced peak in the *SAR* as the particle size increases, followed by a decline beyond an optimal size, indicating a size-dependent resonance effect. Figure 3c,d provide two-dimensional representations, revealing that *SAR* enhancement strongly correlates with field frequency and amplitude. The highest *SAR* values are near 20 nm, suggesting that this size optimally balances Néel and Brownian relaxation mechanisms [37,38]. At lower field strengths and frequencies, the *SAR* response is significantly reduced, emphasizing the critical role of both parameters in optimizing hyperthermia performance [39,40]. These findings demonstrate the strong interplay between particle size, field conditions, and heating efficiency in LSMO nanoparticles, offering insights into their potential application in magnetic hyperthermia.

Figure 3a,c show that the *SAR* increases nonlinearly as *H* increases. There is a specific value of particle size for a fixed frequency where energy dissipation is most efficient. Also, the SAR increases significantly with the frequency. A resonant peak in the *SAR* appears around 18 nm (Figure 3b,d).

**Figure 4:**
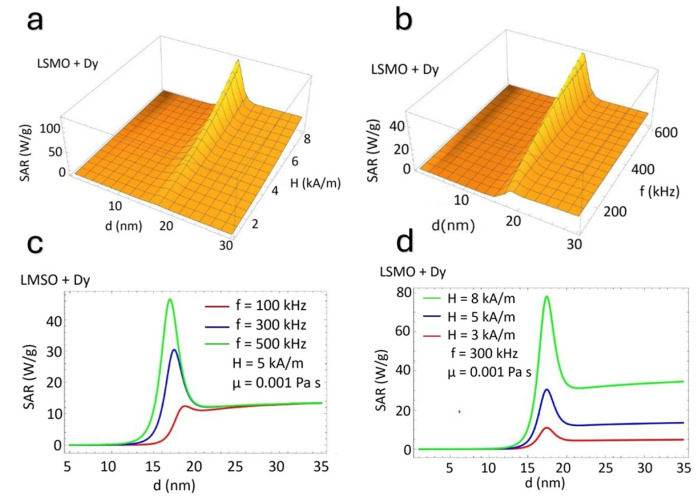
(**a**,**b**) Three-dimensional surface plots and (**c**,**d**) two-dimensional contour plots of the *SAR* (W/g) for Dy-doped LSMO nanoparticles as a function of size (*d*, nm), field strength (*H*, KA/m), and frequency (*f*, kHz). Note the reduced optimal particle size and shifted *SAR* peak due to Dy substitution.

Figure 4 presents the dependence of the SAR on the nanoparticle size (d) for Dy-doped (LSMO + Dy) nanoparticles under varying magnetic field amplitudes and frequencies. Similar to undoped LSMO, the *SAR* peaks at an optimal nanoparticle size (d ≈ 18 nm), followed by a decline for larger sizes. Figure 4a,b illustrate three-dimensional surface plots, showing a strong dependence on magnetic field amplitude (*H*) and frequency (*f*). Figure 4c,d confirm this trend, demonstrating that the SAR increases with increasing field parameters. However, unlike undoped LSMO (Figure 3), Dy incorporation results in a lower SAR magnitude across all conditions. A direct comparison between Figure 3 and Figure 4 reveals the influence of Doping Dy on the *SAR* of LSMO nanoparticles. In both cases, the SAR strongly depends on nanoparticle size, magnetic field amplitude, and frequency, with a distinct peak at an optimal particle size. However, a notable shift is observed: while undoped LSMO (Figure 3) reaches the maximum *SAR* at approximately d ≈ 20 nm, Dy-doped LSMO (Figure 4) exhibits a reduced optimal size around *d* ≈ 18 nm. This shift suggests that Dy incorporation alters the magnetic anisotropy energy, influencing the balance between Néel and Brownian relaxation mechanisms. Moreover, Dy doping significantly reduces the overall *SAR* magnitude. LSMO exhibits higher SAR values than LSMO + Dy with identical field parameters, indicating a suppression of magnetic energy dissipation upon Dy substitution. This reduction is attributed to an increase in the magnetic anisotropy barrier, which affects the adequate relaxation time and limits the efficiency of hyperthermia heating [41,42]. Despite maintaining a similar trend in *SAR* enhancement with increasing H and f, the presence of Dy systematically lowers the heating efficiency. These findings underscore the crucial role of rare-earth doping in tuning the magnetic and thermal properties of LSMO nanoparticles, providing insights for the optimization of materials for magnetic hyperthermia applications [43].

Figure 4 describes the behavior of *SAR* values for Dy-doped LSMO. Lower *SAR* values can be observed after Dy doping. The resonance (SAR peak) appears (similar to undoped LSMO) for a specific particle diameter (~15–16 nm). The maximum value of the *SAR* (SAR peak) describes the resonance between the magnetic relaxation mechanism (Néel and Brownian mechanisms) and applied magnetic field parameters (frequency and magnetic amplitude). Dy doping influences the magnetic anisotropy (*K*) and, implicitly, the Néel relaxation time ($\tau_{N}$). The energy barrier for the Néel relaxation increases when the magnetocrystalline anisotropy (*K*) increases as a result of doping. Therefore, the increase in the Néel time ($\tau_{N})$ determines the shift of the SAR peak to a smaller particle size. The decrease in the SAR can be attributed to slower relaxation dynamics [44,45].

**Figure 5:**
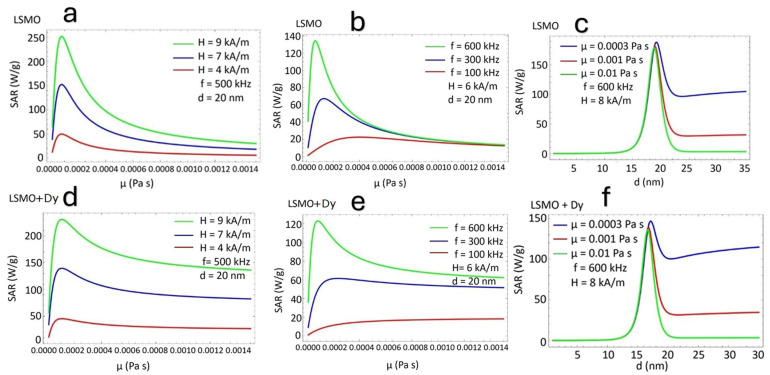
(**a**,**b**) SAR (W/g) of LSMO and (**d**,**e**) Dy-doped LSMO nanoparticles as a function of ferrofluid viscosity (μ, Pa·s). (**c**,**f**) SAR versus particle size (d, nm) and viscosity.

Figure 5 presents the dependence of the SAR on the ferrofluid viscosity (μ) for both (LSMO) and Dy-doped (LSMO + Dy) nanoparticles under varying magnetic field amplitudes and frequencies. The results demonstrate the strong sensitivity of the *SAR* to viscosity, highlighting the interplay between Brownian and Néel relaxation mechanisms [46]. In Figure 5a,b, the SAR of LSMO exhibits a non-monotonic trend with increasing viscosity, initially increasing at low viscosities before reaching a peak and subsequently decreasing. This behavior suggests an optimal viscosity range where relaxation dynamics are most efficient. The observed trends indicate that at very low viscosities, Brownian relaxation is dominant, while at higher viscosities, Néel relaxation becomes the primary mechanism [47,48]. A similar pattern is observed in Figure 5d,e for Dy-doped LSMO, though with lower *SAR* values than LSMO. A direct comparison between LSMO and LSMO + Dy reveals key differences. First, Dy doping leads to an overall reduction in the *SAR* across all viscosity ranges, consistent with the previously observed effects of Dy-induced changes in magnetic anisotropy [49,50]. Second, the optimal viscosity for the peak SAR slightly shifts in LSMO + Dy, indicating that Dy incorporation modifies the relaxation time distribution. Figure 5c,f further illustrate the combined effect of viscosity and nanoparticle size, showing that the *SAR* is maximized around 20 nm in LSMO and slightly lower (*d* ≈ 18 nm) in LSMO + Dy. Additionally, at higher viscosities (*μ* = 0.01 Pa·s), the decrease in the SAR is more pronounced in LSMO + Dy, indicating a stronger suppression of Brownian relaxation than in undoped LSMO. These findings highlight the crucial role of ferrofluid viscosity in determining hyperthermia efficiency, with Dy doping significantly influencing relaxation mechanisms. The observed trends provide insights for the optimization of SAR performance through precise viscosity control and magnetic anisotropy, which is critical for applications in magnetic fluid hyperthermia.

The non-monotonic dependence of the *SAR* on viscosity presented in Figure 5 describes the competition between Néel and Brownian relaxation mechanisms. The SAR is maximized when the effective relaxation time (τ) resonates with the frequency of the applied magnetic field. At low viscosities, Brownian relaxation dominates but is too fast (the nanoparticle rotates easily) for efficient heating. In this case, the nanoparticle does not lose energy. This behaviour demonstrates the low values of the SAR. As viscosity increases, rotational motion slows, and the SAR increases. Further increases suppress the Brownian contribution, and the SAR decreases. This crossover point is frequency- and size-dependent. Dy doping increases magnetic anisotropy, slowing Néel relaxation and slightly shifting the crossover while reducing the overall SAR due to magnetic dilution. As a result, the SAR peak becomes sharper and occurs at slightly lower viscosities in Dy-LSMO compared to LSMO.

## 6. Experimental Setup and SAR Measurement Method

The SAR of magnetic nanoparticles in an alternating magnetic field was evaluated using a custom-built experimental setup designed to operate within a magnetic field range of 0 to 100–150 Oe, where the amplitude is the RMS field value, depending on the applied frequency. The system allows for discrete frequency adjustments at 150, 200, 250, and 300 kHz, ensuring precise control over the experimental conditions. The setup consists of four primary components: (i) a magnetic field generation system comprising a power supply, transformer, signal generator, and magnetic coil; (ii) a temperature measurement system equipped with a thermocouple and a voltmeter for real-time thermal monitoring; (iii) a cooling system incorporating a circulating pump and distilled water loop to maintain coil stability; and (iv) an external control system, which includes a personal computer interfaced with a switching device for automated data acquisition and control. For *SAR* measurements, a test tube containing a dispersion of nanoparticles in water was placed at the coil’s central working aperture, ensuring optimal exposure to the region of maximum magnetic field intensity. The magnetic field was applied for a fixed time interval, during which the temperature rise induced by nanoparticle heating was continuously recorded. Following each measurement, thermal parameters were extracted using established analytical models. The control software facilitated precise regulation of the magnetic field application time and the sampling interval for temperature acquisition. *SAR* values were determined using both the Box–Lucas and corrected slope methods. The latter, which provides enhanced accuracy in cases of rapid thermal saturation, accounts for both the heating and cooling curves following the removal of the magnetic field. Specifically, the corrected slope method defines the adiabatic heating power as the sum of the absolute values of the initial heating and post-field cooling slopes, ensuring a robust and reliable quantification of nanoparticle heating efficiency.

**Figure 6:**
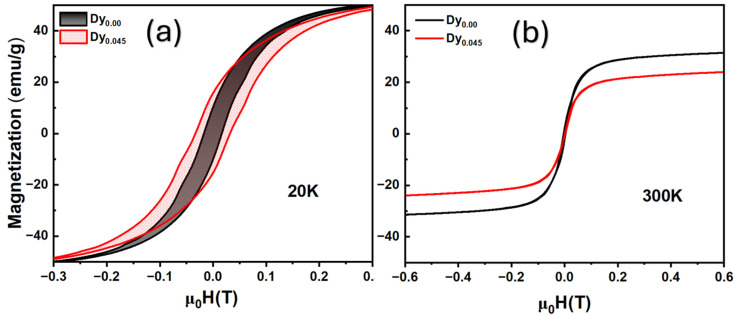
(**a**,**b**) Field-dependent magnetization at 20 K (**a**) and 300 K (**b**) for LSMO and Dy-doped LSMO nanoparticles.

Hysteresis measurement data indicate the absence of hysteresis (Figure 6b), so our particles are superparamagnetic because they should not exhibit hysteresis, theoretically [47]. If we talk about domains, there are three different concepts: a single-domain state (no domain walls in the sample), homogeneous state (the direction of magnetization in the sample is the same at all points), or superparamagnetic state (the direction of the magnetic moment of the particle changes chaotically over time). The radius of the homogeneous state is smaller than the radius of the single-domain state. The radius of the superparamagnetic state is usually the smallest of all, so if we see superparamagnetic hysteresis, then there are good reasons to believe that the particle is in a single-domain state. Through the approximation of the *SAR*(H) by a power function, the exponent was found to be equal to a = 1.81 for Dy-doped LSMO nanoparticles at a frequency of 250 kHz, and for nanoparticles without the addition of dysprosium, the exponent was equal to a = 1.95, which confirms the superparamagnetic state of the nanoparticles because the exponent would be more than 3.0 in the event of hysteresis.

**Figure 7:**
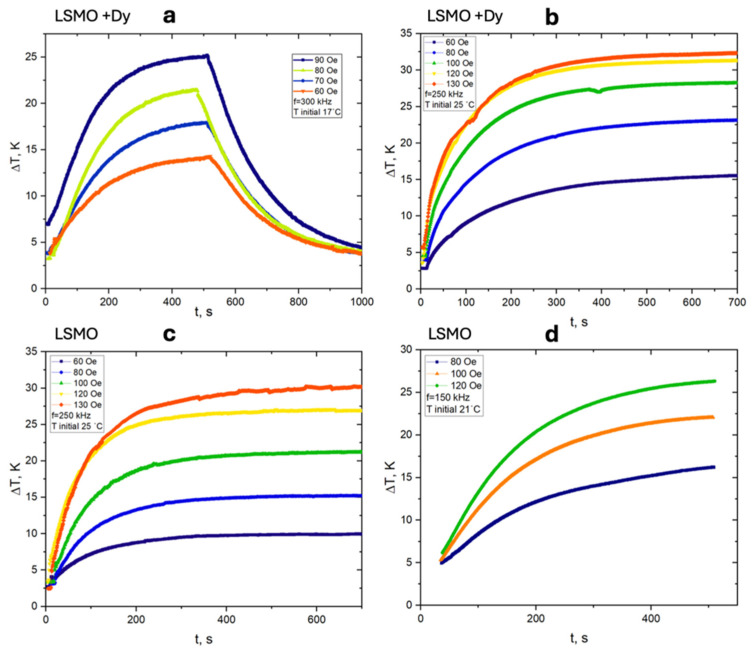
(**a**–**d**) Temperature evolution curves for heating and cooling of LSMO and Dy-doped LSMO nanoparticles under different AC field conditions. (**a**) 300 kHz, 60 to 90 Oe, initial T = 17 °C; (**b**) 250 kHz, 60 to 130 Oe, T = 25 °C; (**c**) 250 kHz, 60 to 90 Oe, T = 25 °C; (**d**) 150 kHz, 60 to 120 Oe, T = 21 °C.

Figure 7 presents the heating and cooling curves for Dy-doped LSMO and LSMO nanoparticles under alternating magnetic fields at different amplitudes and frequencies. The results provide insights into these materials’ heating efficiency and thermal dissipation dynamics, with the SAR values summarized in Table 1 and Table 2. For Dy-doped LSMO (Figure 7a,b), heating curves recorded at 300 kHz and 250 kHz reveal a clear dependence on the applied field strength. At higher field amplitudes, the temperature rise (Δ*T*) is significantly enhanced, reaching a maximum of 25–30 K at 90 Oe. A rapid initial temperature increase is observed, followed by a gradual plateau, indicating thermal equilibrium. The cooling phase exhibits a steady decrease, characteristic of heat dissipation to the surrounding medium. Notably, the heating efficiency improves with increasing field amplitude, as confirmed by Table 1, which shows that *SAR* values rise from 4.87 W/g at 70 Oe to 12.8 W/g at 120 Oe, demonstrating increased power absorption with enhanced field strength. In contrast, the heating curves for undoped LSMO (Figure 7c,d) measured at 250 kHz and 150 kHz exhibit a similar trend but lower overall heating efficiency. The maximum temperature increase remains below 25 K, and the heating rate is slower. The *SAR* values in Table 2 confirm this trend, with lower values across all field amplitudes. At 120 Oe, the *SAR* for LSMO reaches 14.36 W/g, whereas Dy-doped LSMO achieves 12.8 W/g at the same field amplitude and at a 250 kHz frequency, suggesting that Dy substitution alters the magnetic energy dissipation mechanisms. A comparative analysis of Table 1 and Table 2 reveals that SAR values for Dy-doped LSMO are consistently higher at lower field amplitudes and moderate frequencies, whereas LSMO exhibits superior performance at higher field strengths and lower frequencies.

In Dy-doped LSMO, dysprosium (Dy^3+^) modifies the local magnetic interactions, affecting the SAR. Dysprosium has strong spin-orbit coupling and a significant magnetic moment (~10.63 µB) [51]. These characteristics strongly influence its magnetic properties. When LSMO is doped (introduced into LSMO), Dy^3+^ ions increase magnetic anisotropy due to their strong interaction with Mn^3+^/Mn^4+^ moments. Anisotropy may include contributions from magneto crystalline anisotropy, surface disordering, or shape anisotropy. Surface anisotropy dominates at nanoparticle sizes below 10 nanometers. The anisotropy constants generally depend on many external factors and, in the case of magnetic nanoparticles, are not precisely determined experimentally but are distributed around the mean value; as a rule, the corresponding value for the bulk material is taken into consideration in calculations. For this reason, the value of the constant is chosen in the most frequently observed range of *K* = 10^4^ ÷ 10^5^ J⋅m^−3^. Magnetic anisotropy determines the energy barrier for magnetization reversal. Its influence on heating depends on the mechanism. High *K* values increase the coercive field (*H_c_*), enlarging the area of the hysteresis loop and theoretically boosting the SAR. But our particles are single-domain and superparamagnetic, with negligible hysteresis losses. If we speak about Néel relaxation, high anisotropy increases the *τ_N_*, potentially shifting it out of sync with the applied field frequency. If *τ_N_* ≫ 1/*f*, spins cannot relax quickly enough, reducing energy dissipation. In the linear response regime (low fields), the SAR scales with H^2^ for SPM, but high *K* values require stronger fields to achieve magnetization saturation.

Dy insertion also introduces bias effects, leading to a shifted and narrowed hysteresis loop. These narrower loops mean lower energy dissipation per cycle, reducing the SAR. The system reaches an anisotropy energy barrier where further field increases do not contribute to additional heating. Dy introduces localized spin disorder and higher magnetic anisotropy, limiting further magnetization rotation. Dy-LSMO is inferior for hyperthermia at high magnetic fields due to the following aspects: (i) increased anisotropy requires stronger fields to achieve magnetization saturation, and (ii) possible spin-glass formation efficient dipolar interactions. Overall, these findings indicate that both LSMO and Dy-doped LSMO exhibit field- and frequency-dependent heating behavior, with Dy doping affecting the energy dissipation efficiency. The results suggest that optimizing the frequency and field amplitude is crucial for maximizing the SAR in these manganite-based nanoparticles, which have potential applications in magnetic hyperthermia and other energy-related technologies. Among the possible explanations for the reduced SAR in Dy-doped LSMO at high magnetic fields, one hypothesis involves the onset of spin glass-like behavior. Incorporating Dy^3+^ increases local magnetic anisotropy and introduces random interactions due to size distribution and surface spin disorder. Such conditions can promote magnetic frustration, creating a spin glass-like state where magnetic moments are frozen in random orientations. In this disordered state, the long-range alignment of magnetic dipoles is disrupted, weakening effective dipole–dipole interactions between nanoparticles. Since these dipolar interactions can enhance magnetic relaxation and energy dissipation under alternating magnetic fields, their suppression reduces collective heating efficiency, contributing to the observed drop in the *SAR* at high field amplitudes.

## 7. Conclusions

In summary, we have demonstrated the tunable magnetic heating behavior of LSMO and Dy-doped LSMO nanoparticles as a function of magnetic field amplitude and frequency. SAR values exhibit a non-monotonic dependence on particle size, with Dy incorporation leading to reduced heating efficiency due to enhanced magnetic anisotropy and altered relaxation dynamics. To ensure experimental consistency, it is recommended to begin heating measurements from the same initial temperature and avoid prolonged delays between tests to prevent nanoparticle aggregation or sticking effects. Frequency-dependent studies confirm that the SAR follows a power-law relationship, with Dy doping shifting the optimal particle size toward smaller diameters. Additionally, theoretical modeling based on Néel and Brownian relaxation mechanisms was used to explore the impact of ferrofluid viscosity on the SAR, highlighting the complex interplay between rotational and magnetic relaxation processes. Although no direct experimental viscosity-dependent measurements were performed, these simulations emphasize the importance of fluidic properties in optimizing heating performance. Overall, our findings provide valuable insights into the design and optimization of perovskite-based manganite nanoparticles for controlled magnetic hyperthermia and other energy-related applications.

## Figures and Tables

**Table 1:** SAR values at different frequencies and field amplitudes for Dy-Doped LSMO nanoparticles.

f, kHz	H, Oe	70	80	90	100	120
150			5.85		7.23	9.0
250			7.01		10.48	12.8
300		4.87	6.88	8.49		

**Table 2:** SAR values at different frequencies and field amplitudes for LSMO nanoparticles.

f, kHz	H, Oe	70	80	90	100	120
150			4.0		6.78	7.85
250			6.71		9.76	14.36
300		2.93	3.73	4.05		

## Data Availability

Data is contained within the article.

## References

[1] Gavilán H., Avugadda S.K., Fernández-Cabada T., Soni N., Cassani M., Mai B.T., Chantrell R., Pellegrino T. (2021). Magnetic nanoparticles and clusters for magnetic hyperthermia: Optimizing their heat performance and developing combinatorial therapies to tackle cancer. Chem. Soc. Rev..

[2] Carter T.J., Agliardi G., Lin F.-Y., Ellis M., Jones C., Robson M., Richard-Londt A., Southern P., Lythgoe M., Thin M.Z. (2021). Potential of Magnetic Hyperthermia to Stimulate Localized Immune Activation. Small.

[3] Tay Z.W., Chandrasekharan P., Chiu-Lam A., Hensley D.W., Dhavalikar R., Zhou X.Y., Yu E.Y., Goodwill P.W., Zheng B., Rinaldi C. (2018). Magnetic Particle Imaging-Guided Heating in Vivo Using Gradient Fields for Arbitrary Localization of Magnetic Hyperthermia Therapy. ACS Nano J..

[4] Cazares-Cortes E., Cabana S., Boitard C., Nehlig E., Griffete N., Fresnais J., Wilhelm C., Abou-Hassan A., Ménager C. (2019). Recent insights in magnetic hyperthermia: From the “hot-spot” effect for local delivery to combined magneto-photo-thermia using magneto-plasmonic hybrids. Adv. Drug Deliv. Rev..

[5] Brezovich I.A., Meredith R.F. (1989). Practical aspects of ferromagnetic thermoseed hyperthermia. Radiol. Clin. N. Am..

[6] Ma Z., Mohapatra J., Wei K., Liu J.P., Sun S. (2023). Magnetic Nanoparticles: Synthesis, Anisotropy, and Applications. Chem. Rev..

[7] Barrera G., Allia P., Tiberto P. (2020). Temperature-dependent heating efficiency of magnetic nanoparticles for applications in precision nanomedicine. Nanoscale.

[8] Lavorato G.C., Das R., Masa J.A., Phan M.-H., Srikanth H. (2021). Hybrid magnetic nanoparticles as efficient nanoheaters in biomedical applications. Nanoscale Adv..

[9] Cardoso V.F., Francesko A., Ribeiro C., Bañobre-López M., Martins P., Lanceros-Mendez S. (2018). Advances in Magnetic Nanoparticles for Biomedical Applications. Adv. Healthc. Mater..

[10] Li Z., Li X., Zong Y., Tan G., Sun Y., Lan Y., He M., Ren Z., Zheng X. (2017). Solvothermal synthesis of nitrogen-doped graphene decorated by superparamagnetic Fe_3_O_4_ nanoparticles and their applications as enhanced synergistic microwave absorbers. Carbon.

[11] Zhao W., Liu Z., Sun Z., Zhang Q., Wei P., Mu X., Zhou H., Li C., Ma S., He D. (2017). Superparamagnetic enhancement of thermoelectric performance. Nature.

[12] Faílde D., Ocampo-Zalvide V., Serantes D., Iglesias Ò. (2024). Understanding magnetic hyperthermia performance within the “Brezovich criterion”: Beyond the uniaxial anisotropy description. Nanoscale.

[13] Yan Y., Li Y., You J., Shen K., Chen W., Li L. (2025). Morphology-dependent magnetic hyperthermia characteristics of Fe_3_O_4_ nanoparticles. Mater. Chem. Phys..

[14] Dizajyekan B.S., Jafari A., Vafaie-Sefti M., Saber R., Fakhroueian Z. (2024). Preparation of stable colloidal dispersion of surface modified Fe_3_O_4_ nanoparticles for magnetic heating applications. Sci. Rep..

[15] Getahun Y., Habib A., Erives-Sedano V., Lee W.-Y., Poon W., El-Gendy A.A. (2024). Superparamagnetic iron oxide nanoparticles functionalized by biocompatible ligands with enhanced high specific absorption rate for magnetic hyperthermia. Colloids Surf. A Physicochem. Eng. Asp..

[16] Pimentel B., Caraballo-Vivas R.J., Checca N.R., Zverev V.I., Salakhova R.T., Makarova L.A., Pyatakov A.P., Perov N.S., Tishin A.M., Shtil A.A. (2018). Threshold heating temperature for magnetic hyperthermia: Controlling the heat exchange with the blocking temperature of magnetic nanoparticles. J. Solid State Chem..

[17] Mamiya H., Jeyadevan B. (2011). Hyperthermic effects of dissipative structures of magnetic nanoparticles in large alternating magnetic fields. Sci. Rep..

[18] Mille N., Faure S., Estrader M., Yi D., Marbaix J., De Masi D., Soulantica K., Millán A., Chaudret B., Carrey J. (2021). A setup to measure the temperature-dependent heating power of magnetically heated nanoparticles up to high temperature. Rev. Sci. Instrum..

[19] Xu X., Xie W., Li F., Niu C., Li M., Wang H. (2024). General approach for efficient prediction of refrigeration performance in caloric materials. Phys. Rev. Appl..

[20] Vajtai L., Nemes N.M., Morales M.D., Molnár K., Pinke B.G., Simon F. (2024). Incidence of the Brownian Relaxation Process on the Magnetic Properties of Ferrofluids. Nanomaterials.

[21] Mansour M., Sedky A., Alshammari A.S., Khan Z.R., Bouzidi M., Alshammari M.S. (2024). Structural, Optical, Magnetic, and Dielectric Investigations of Pure and Co-Doped La_0.67_Sr_0.33_Mn_1−x−y_ZnxCoyO_3_ Manganites with (0.00 < x + y < 0.20). Crystals.

[22] Sharma R., Kumar P., Anil A. (2024). Insights into structural and magnetic properties of Lanthanum Manganite ceramic co-doped with calcium and cobalt. Preprints.

[23] Ramlan F.H., Setiawan J., Susetyo F.B., Akbar H. (2024). Preparation, Synthesis and Characterization of La (1−x) Sr (x) MnO_3_ Alloy. J. Appl. Eng. Technol. Sci..

[24] Brahem R. (2024). LSNMTix Contribution to the examination of the effect of Ti–substitution on the crystallographic and transport characteristics of. J. Qassim Univ. Sci..

[25] Zaidi N., Mnefgui S., Dhahri A., Dhahri J., Hlil E.K. (2014). The effect of Dy doped on structural, magnetic and magnetocaloric properties of La_0.67−x_Dy_x_Pb_0.33_MnO_3_ (x = 0.00, 0.15 and 0.20) compounds. Phys. B Condens. Matter.

[26] Manna P., Kanthal S., Aquilanti G., Banerjee A., Bandyopadhyay S. (2022). Correlated temperature and field dependent magnetization: Enhanced magnetic hysteresis upon Dy doping in La-based francisite Cu_3_La(SeO_3_)_2_O_2_Cl. J. Magn. Magn. Mater..

[27] Ribeiro J.L., Vieira L.G. (2010). Landau model for the phase diagrams of the orthorhombic rare-earth manganites RMnO_3_ (R = Eu, Gd, Tb, Dy, Ho). Phys. Rev. B.

[28] Zhang Y., Liu F., Zheng T., Zhang Z., Liu W., Zhao X., Liu X. (2015). Synthesis of perovskite-type manganites Yb_1−x_Dy_x_MnO_3_ (0.1 ≤ x ≤ 0.5) via solid-state reaction and high-pressure flux methods followed by structural characterization and magnetic property studies. New J. Chem..

[29] Chakraborty A.R., Toma F.T.Z., Alam K., Yousuf S.B., Hossain K.S. (2024). Influence of annealing temperature on Fe_2_O_3_ nanoparticles: Synthesis optimization and structural, optical, morphological, and magnetic properties characterization for advanced technological applications. Heliyon.

[30] Attanayake S.B., Chanda A., Das R., Phan M.H., Srikanth H. (2023). Effects of annealing temperature on the magnetic properties of highly crystalline biphase iron oxide nanorods. AIP Adv..

[31] Smari M., Hamdi R., Mansour S.A., Al-Haik M.Y., Zakaria Y., Haik Y. (2025). Dy-Doped La_0.51_Sr_0.49_MnO_3_ nanoparticles: Tuning structural and magnetocaloric properties via Sol-Gel synthesis for energy-efficient applications. Nano Trends.

[32] Astefanoaei I., Gimaev R., Zverev V., Tishin A., Stancu A. (2023). Cubic and Sphere Magnetic Nanoparticles for Magnetic Hyperthermia Therapy: Computational Results. Nanomaterials.

[33] Ferreira M.C., Pimentel B., Andrade V., Zverev V., Gimaev R.R., Pomorov A.S., Pyatakov A., Alekhina Y., Komlev A., Makarova L. (2021). Understanding the Dependence of Nanoparticles Magnetothermal Properties on Their Size for Hyperthermia Applications: A Case Study for La-Sr Manganites. Nanomaterials.

[34] Astefanoaei I., Gimaev R., Zverev V., Stancu A. (2019). Modelling of working parameters of Gd and FeRh nanoparticles for magnetic hyperthermia. Mater. Res. Express.

[35] Kahil H., Faramawy A., El-Sayed H., Abdel-Sattar A. (2021). Magnetic Properties and SAR for Gadolinium-Doped Iron Oxide Nanoparticles Prepared by Hydrothermal Method. Crystals.

[36] Shayestefar M., Mirahmadi-Zare S.Z., Mashreghi A., Hasani S. (2025). Investigation of magnetic and structural properties of Dy-substituted Mn-Zn ferrite nanoparticles for hyperthermia applications. J. Sol-Gel Sci. Technol..

[37] Shah R.R., Davis T.P., Glover A.L., Nikles D.E., Brazel C.S. (2015). Impact of magnetic field parameters and iron oxide nanoparticle properties on heat generation for use in magnetic hyperthermia. J. Magn. Magn. Mater..

[38] Xin S., Sun J., Shi Z., Li R., Liu X., Wang N., Weaver J.B., Wu K. (2025). Study and optimization on hyperthermia performance of magnetic fluids modeled by coupled Brownian–Néel rotations. J. Appl. Phys..

[39] Egea-Benavente D., Ovejero J.G., Morales M.D., Barber D.F. (2021). Understanding MNPs Behaviour in Response to AMF in Biological Milieus and the Effects at the Cellular Level: Implications for a Rational Design That Drives Magnetic Hyperthermia Therapy toward Clinical Implementation. Cancers.

[40] Singh A., Kumar P., Pathak S., Jain K., Garg P., Pant M., Mahapatro A.K., Rath D., Wang L., Kim S.-K. (2023). A threefold increase in SAR performance for magnetic hyperthermia by compositional tuning in zinc-substituted iron oxide superparamagnetic nanoparticles with superior biocompatibility. J. Alloys Compd..

[41] Thorat N.D., Khot V.M., Salunkhe A.B., Prasad A.I., Ningthoujam R.S., Pawar S.H. (2013). Surface functionalized LSMO nanoparticles with improved colloidal stability for hyperthermia applications. J. Phys. D Appl. Phys..

[42] McBride K., Cook J., Gray S., Felton S., Stella L., Poulidi D. (2016). Evaluation of La1−xSrxMnO_3_ (0 ≤ x < 0.4) synthesised via a modified sol–gel method as mediators for magnetic fluid hyperthermia. CrystEngComm.

[43] Asghar M.S., Ghazanfar U., Rizwan M., Manan M.Q., Baig A., Qaiser M.A., Haq Z., Wang L., Duta L. (2025). Potential Molecular Interactions and In Vitro Hyperthermia, Thermal, and Magnetic Studies of Bioactive Nickel-Doped Hydroxyapatite Thin Films. Int. J. Mol. Sci..

[44] Tanabe K., Maekawa Y., Wada H., Yamauchi K., Oguchi T., Harima H. (2022). Hall effect of itinerant electron metamagnet Co(S1-xSex)_2_. J. Magn. Magn. Mater..

[45] Aqra F. (2014). The cohesive energy density and the isothermal compressibility: Their relationships with the surface tension. Phys. B Condens. Matter..

[46] Devi Y.H., Singh L.H., Wareppam B., Swain B.P. (2022). Effects of Viscosity on the Magnetic-Induced Heat Generation BT—Advances in Nanostructured Materials. Advances in Nanostructured Materials.

[47] Viktorov V., Nimafar M. (2013). A novel generation of 3D SAR-based passive micromixer: Efficient mixing and low pressure drop at a low Reynolds number. J. Micromechanics Microengineering.

[48] Jiang C., Guo L., Li Y., Li S., Tian Y., Ma L., Luo J. (2021). Magnetic field effect on apparent viscosity reducing of different crude oils at low temperature. Colloids Surf. A Physicochem. Eng. Asp..

[49] Yin L., Zhang S., Sun M., Wang S., Huang B., Du Y. (2023). Heteroatom-Driven Coordination Fields Altering Single Cerium Atom Sites for Efficient Oxygen Reduction Reaction. Adv. Mater..

[50] Laha S.S., Thorat N.D., Singh G., Sathish C.I., Yi J., Dixit A., Vinu A. (2022). Rare-Earth Doped Iron Oxide Nanostructures for Cancer Theranostics: Magnetic Hyperthermia and Magnetic Resonance Imaging. Small.

[51] Sibanda E.T., Prinsloo A.R.E., Sheppard C.J., Mohanty P. (2022). Structural and magnetic properties of DyCrO_3_. AIP Adv..

